# A 13-year termite (Insecta, Blattodea) monitoring programme in the Azores: Dataset and findings

**DOI:** 10.3897/BDJ.13.e164834

**Published:** 2025-10-23

**Authors:** Paulo A.V. Borges, Sónia Bettencourt, Dejalme Vargas, Raquel Medeiros, João Melo, Ana Rodrigues

**Affiliations:** 1 University of Azores, CE3C—Centre for Ecology, Evolution and Environmental Changes, Azorean Biodiversity Group, CHANGE —Global Change and Sustainability Institute, School of Agricultural and Environmental Sciences, Rua Capitão João d’Ávila, Pico da Urze, 9700-042, Angra do Heroísmo, Azores, Portugal University of Azores, CE3C—Centre for Ecology, Evolution and Environmental Changes, Azorean Biodiversity Group, CHANGE —Global Change and Sustainability Institute, School of Agricultural and Environmental Sciences, Rua Capitão João d’Ávila, Pico da Urze, 9700-042 Angra do Heroísmo, Azores Portugal; 2 IUCN SSC Atlantic Islands Invertebrate Specialist Group, Angra do Heroísmo, Azores, Portugal IUCN SSC Atlantic Islands Invertebrate Specialist Group Angra do Heroísmo, Azores Portugal; 3 IUCN SSC Monitoring Specialist Group, Angra do Heroísmo, Azores, Portugal IUCN SSC Monitoring Specialist Group Angra do Heroísmo, Azores Portugal; 4 Secretaria Regional do Ambiente e Acão Climática, Direção Regional do Ambiente e Ação Climática, Rua Cônsul Dabney, Colónia Alemã, Apartado 140, 9900-014, Horta, Azores, Portugal Secretaria Regional do Ambiente e Acão Climática, Direção Regional do Ambiente e Ação Climática, Rua Cônsul Dabney, Colónia Alemã, Apartado 140, 9900-014 Horta, Azores Portugal

**Keywords:** Azores Archipelago, termite monitoring, invasive termites, long-term dataset, urban pests.

## Abstract

**Background:**

From 2011 to 2024, the Azorean Government tested two coordinated monitoring programmes across the archipelago to survey four invasive termite species: the West Indian drywood termite, *Cryptotermes brevis* (Walker, 1853); the yellow-necked drywood termite, *Kalotermes flavicollis* (Fabricius, 1793); the Western European subterranean termite, *Reticulitermes grassei* Clément, 1978; and the eastern subterranean termite, *Reticulitermes flavipes* (Kollar, 1837). The monitoring programme was mostly directed to the detection of *C. brevis* in new locations. Drywood species were detected on multiple islands, with *C. brevis* established on six islands (from west to east: Faial, Pico, São Jorge, Terceira, São Miguel and Santa Maria) and exhibiting the highest infestation densities in the urban centres of the three most important islands in terms of economic activity and human population (São Miguel, Terceira and Faial). *Kalotermes flavicollis* occurs more sporadically, primarily along the south coasts of Terceira, São Miguel and southeast coast of Faial and seldom attains the pest status of *C. brevis*. In contrast, the two *Reticulitermes* species remain restricted to localised subterranean infestations: *Reticulitermes grassei* in Horta (Faial) and *R. flavipes* near Lajes Air Force Base (Terceira), each detected via house inspection visits. Collectively, these efforts provide the first comprehensive, archipelago-wide dataset on termite presence, laying the groundwork for targeted Integrated Pest Management strategies in the Azores.

**New information:**

Records of *Cryptotermes brevis* overwhelmingly dominated the monitoring data, comprising 1,801 of the 1,832 total events (98%), a pattern consistent with previous surveys of its rapid spread in the Azorean urban environment. These detections were heavily concentrated on two islands: Terceira (n = 919) and São Miguel (n = 755). In contrast, Faial, Pico and Santa Maria each yielded roughly 40 records and São Jorge only seven. Annual trap‐capture counts across all islands increased steadily from approximately 40 captures in 2011 to 154 in 2024, peaking at 185 in 2023.

*Kalotermes flavicollis* was the second most frequently recorded species (n = 24), with most records originating on São Miguel, mirroring its more restricted distribution. The two subterranean termites, *Reticulitermes grassei* and *R. flavipes*, were documented exclusively on Faial and Terceira, respectively, consistent with their historically limited foothold in the archipelago.

Now established on every surveyed island and exhibiting an upward trajectory in annual detection counts, *C. brevis* remains the foremost urban termite threat in the Azores. To forestall further structural outbreaks, Integrated Pest Management should place sustained emphasis on early detection — through year-round trap checks — and on heightened public awareness, by encouraging residents to report both the characteristic pin-sized faecal pellets and any termite occurrence observed during swarming periods.

## Introduction

Termites play a central role in tropical and subtropical ecosystems, driving decomposition of lignocellulosic material, enhancing soil structure and fertility and facilitating nutrient turnover (Fox-Dobbs et al. 2010, Jouquet et al. 2011). Indeed, termites are frequently described as ecosystem engineers due to their impact on soil structure and nutrient distribution (Jouquet et al. 2011, Lavelle et al. 2021).

Despite these benefits, several native (autochthonous) and invasive termite species have become major urban pests, causing structural damage that costs billions of euros annually and necessitates costly control measures (Buczkowski and Bertelsmeier 2017). Termites are causing extensive damage to buildings, wooden structures and furniture by feeding on cellulose‐based materials, including framing timbers, flooring, door and window frames and even decorative veneers (Ridout 2023). In addition to direct structural harm, termite galleries facilitate moisture ingress and fungal decay, further accelerating wood degradation and increasing repair costs (Martín and López 2023).

Islands are especially susceptible to termite introductions, since reduced native termite diversity and limited natural enemies often allow colonisers to proliferate unchecked once established (Austin et al. 2012, Su and Scheffrahn 2000). In the Azores Archipelago, four invasive termites are currently known (Borges et al. 2022): the West Indian drywood termite, *Cryptotermes brevis* (Walker, 1853); the yellow-necked drywood termite, *Kalotermes flavicollis* (Fabricius, 1793); the Western European subterranean termite, *Reticulitermes grassei* Clément, 1978; and the eastern subterranean termite, *Reticulitermes flavipes* (Kollar, 1837). Recent work revealed rapid spread of *C. brevis* across several islands and localised subterranean infestations of *Reticulitermes* spp. (Austin et al. 2012, Ferreira et al. 2013, Borges et al. 2014). The West Indian drywood termite *C. brevis* is the most destructive drywood species in the Azores, able to tolerate low moisture and to establish hundreds of small colonies within a single piece of timber (Ferreira et al. 2013, Borges et al. 2014, Guerreiro et al. 2014). The Western European subterranean termite *R. grassei* is, so far, confined to the City of Horta (Faial Island), where it builds extensive soil nests and can form multinucleate colonies comprising millions of individuals (Duarte et al. 2018, Hernández-Teixidor et al. 2024). The eastern subterranean termite *R. flavipes* (Kollar, 1837) is restricted, so far, to Terceira Island (Austin et al. 2012). While *K. flavicollis* remains largely restricted to moist heartwood in living trees, *C. brevis* thrives in low-moisture, seasoned timber, often penetrating deeply and causing severe damage to structural elements, floors and furnishings (Ferreira et al. 2013). In contrast, both *R. flavipes* and *R. grassei* forage subterraneously, locating and exploiting isolated wood through soil‐borne galleries and feature a high proportion of neotenic reproductives that enhance their invasive potential (Austin et al. 2012, Duarte et al. 2018).

In response to mounting threats against built heritage and private residences, the Azorean regional government and research teams at the University of the Azores have, over the past two decades, implemented systematic monitoring and control programmes (e.g. TermoDisp, TERMIPAR), pilot chemical wood treatments and fumigation trials (Borges and Myles 2007, Ferreira et al. 2013, Guerreiro et al. 2014).

## General description

### Purpose

To present the results of the Azorean Government termite monitoring carried out in the Azores between 2011 and 2024.

### Additional information

From 2011 through 2024, the Regional Government of the Azores has sustained and expanded two island-wide, multi-species termite‐monitoring initiatives (Secretaria Regional do Ambiente e Alterações Climáticas 2023, Secretaria Regional do Ambiente e Ação Climática 2024):

Infestation Inspection Certificates (SCIT)

Since 2011, every home, commercial building or public facility offered for sale or rent in designated “at‐risk” parishes must present a valid Termite Infestation Inspection Certificate (“SCIT”). Trained technicians — graduates of the University of the Azores’ dedicated pest-inspection course — survey interior woodwork (floors, door‐ and window‐frames, skirting boards), attics and crawl-spaces for six key signs of infestation (live insects, alate wings, mud tubes, frass, damage galleries and feeding holes). Results are logged centrally, driving annual updates of geographic risk maps used by municipalities to target preventative treatments and to inform prospective buyers.

Chromotropic Light Trap Network

In spring 2022, the Government launched a dispersal‐flight surveillance system across all nine islands. Over 1,200 yellow sticky traps (45 × 24 cm) were installed on street‐lights, public parks and private‐property exteriors, attracting alates of the four invasive termite species during their evening swarms (May–September). Each trap was serviced by certified field teams, with catch counts uploaded via mobile app; these data were fed into GIS to reveal new infestation foci, colony density hotspots and year‐to‐year spread dynamics.

## Project description

### Title

Monitoring Termites in the Azores Archipelago: A Comprehensive Dataset (2011–2024).

### Personnel

Species recorded by: Adalberto Borges Couto; Aida de Fátima Brasil Vieira; Ana Casals; Ana Margarida Moniz Soares; Aniceto A. Cordeiro; Annabella Borges; Dejalme Vargas; Emanuel Duarte Costa; Fábio André Azevedo Cerqueira; Fernando Eduardo Costa e Silva; Filipa Vasconcelos da Ponte Valadão Garrett; Filomena Tavarela Ferreira; Francisco Fernandes Lopes; Francisco Mota Vieira Rodrigues da Câmara; Gil da Silva Navalho; Jaime B. Bairos; Joana Correia Soares; Joana Pombo Sousa Tavares; João Luís de Oliveira Pacheco; João M. Sousa; João Manuel Correia Pimentel; João Pedro Martins Gonçalves; João Pedro Pedroso de Lima Ferreira de Matos; Jorge Manuel Lopes Amorim da Cunha; Kenny F. Alves; Lisandra Câmara Miranda; Luís Miguel Gomes Vieira; Luís Miguel Resendes Fernandes Bettencourt da Silva; Luís Miguel Taraio dos Santos Antunes; Luís Paulo Ramalho da Silva Garcia; Manuel António Braga Pinheiro; Manuel José Gonçalves Goulart de Sequeira; Maria L.P.C.B.M. Sequeira; Marina Ponciano Lima; Mauro José Silva Lourenço; Miguel da Cunha Pacheco Ribeiro de Borba; Nélson Simas; Nuno Filipe Ferreira Bicudo da Ponte; Nuno Marco Botelho Soares; Orlando Guerreiro; Paulo Alão Nunes de Sousa; Paulo Alexandre Vilela Martins Raimundo; Pedro Fernando Gonçalves Ribeiro; Pedro Manuel Parreira Brito Do Rio; Pedro Medeiros; Pedro Miguel Machado da Silveira; Rodrigo Cordeniz Ferreira; Rui Filipe Mota Dutra; Rui Manuel de Jesus Martins; Sérgio Cardeira; Tatiana Branco; Tiago M. Silva; Vanessa C.B. Mendonça.

Species Identification: Adalberto Borges Couto; Aida de Fátima Brasil Vieira; Ana Casals; Dejalme Vargas; Emanuel Duarte Costa; Fábio André Azevedo Cerqueira; Filipa Vasconcelos da Ponte Valadão Garrett; Jaime B. Bairos; Joana Pombo Sousa Tavares; Jorge Manuel Lopes Amorim da Cunha; João Luís de Oliveira Pacheco; Lisandra Câmara Miranda; Luís Miguel Gomes Vieira; Luís Paulo Ramalho da Silva Garcia; Manuel António Braga Pinheiro; Manuel José Gonçalves Goulart de Sequeira; Maria LPCBM. Sequeira; Miguel da Cunha Pacheco Ribeiro de Borba; Nélson Simas; Nuno Filipe Ferreira Bicudo da Ponte; Orlando Guerreiro; Paulo Alão Nunes de Sousa; Pedro Fernando Gonçalves Ribeiro; Pedro Medeiros; Pedro Miguel Machado da Silveira; Rui Filipe Mota Dutra; Rui Manuel de Jesus Martins; Sérgio Cardeira; Tatiana Branco.

Data acquisition: Secretaria Regional do Ambiente e Ação Climática (SRAAC).

Darwin Core Database: Paulo A.V. Borges.

### Study area description

The Azores are a Portuguese archipelago of nine volcanic islands stretching over 600 km in the mid-North Atlantic (37–40° N, 25–31° W), roughly 1600 km west of mainland Portugal. Geologically they lie along the Mid-Atlantic Ridge, rising abruptly from the ocean floor to summits over 2,300 m (Mount Pico, 2,351 m) and are grouped into three groups of islands from west to east (Fig. 1): Flores (143 km²) and Corvo (17 km²) (Western Group); Faial (173 km²), Pico (447 km²), São Jorge (237 km²), Graciosa (60 km²), Terceira (401 km²) (Central group); and São Miguel (744 km²) and Santa Maria (97 km²) (Eastern group).

The climate is mild maritime–subtropical, with year-round high humidity, average temperatures of 14–25°C and persistent “Azores High” anticyclones in summer. Volcanism remains active (e.g. Capelinhos 1957–58) and frequent seismicity reflects ongoing rift and fault dynamics.

The archipelago’s largest city and administrative capital is Ponta Delgada (São Miguel, ~ 68,000 inhabitants), followed by Angra do Heroísmo (Terceira, ~ 35,000 inhabitants) and Horta (Faial, ~ 14,000 inhabitants).

### Design description

The Azorean termite surveillance combined two complementary sampling schemes:

Chromotropic‐Trap Network

Trap type and placement: Yellow, chromotropic sticky‐card traps (45 × 24 cm) affixed to public street‐lights and luminaires in 121 parish of the nine islands.

Deployment density: Traps were allocated by island in proportion to the previous knowledge of termite extent of occurrence (e.g. São Miguel 401, Terceira 300, São Jorge 151 etc.) and sited to maximise coverage of both urban cores and peri‐urban fringe zones.

SCIT (Infestation Inspection Certificate) System

Mandatory inspections: In every parish where the sale or rental of a building requires a termite-inspection certificate (Resolution no. 219/2021), trained technicians certified by the University carried out on-site surveys of interior woodwork, crawlspaces and attics. However, any homeowner may also request an inspection at any time and for any purpose.

Spatial analysis: Each confirmed infestation point was georeferenced and fed into a GIS‐based risk‐mapping protocol. Drywood‐termite risk zones were delineated using 100 m buffers around infested structures, whereas subterranean‐termite feeding zones used 500 m buffers (with further subdivisions at 25 m and 50 m radii) to capture colony foraging areas.

Together, these two designs — an archipelago‐wide, systematically serviced trap network and a legally mandated, spatially explicit building‐inspection regimen — ensure robust, year‐round detection of both dispersing alates and established infestations across the Azores.

## Sampling methods

### Study extent

The monitoring and certification surveys were conducted across the entire Autonomous Region of the Azores, encompassing all nine islands and every municipality. Two complementary sampling frames were defined:

Chromotropic‐Trap Network

Between spring and autumn 2022 and 2023, a total of 1,314 yellow, chromotropic sticky traps were deployed outdoors on public luminaires and street-lights to intercept alate termites. Trap allocation by island was as follows: Terceira (300), São Miguel (401), São Jorge (151), Santa Maria (92), Pico (150), Graciosa (40), Flores (50), Faial (100) and Corvo (30).

SCIT (Infestation Inspection Certificate) Area

In parallel, the study harnessed data from the Regional Termite Infestation Certification System (SCIT). The defined SCIT area includes all civil parishes (“freguesias”) in which the sale or rental of any building legally requires presentation of a valid termite-inspection certificate, per Resolution no. 219/2021 of 16 September 2021. These parishes span the nine islands, ensuring that every locale subject to mandatory building certification is represented in the infestation‐mapping framework.

Together, these two sampling domains — extensive trap coverage across all islands and targeted SCIT‐mandated parishes — provide a robust, island-wide portrait of both subterranean and drywood termite occurrence and risk.

### Quality control

All termite sampling followed standardised methods (see above) and the taxonomy of the four species follows the last checklist of Azorean arthropods (Borges et al. 2022).

The spatial resolution of the SCIT inspection occurrence records was reduced to 353.6 m coordinate uncertainty to safeguard the privacy of homeowners and all precise locality information was removed. Trap data, however, were retained without modification.

Methodological bias – the Chromotropic‐Trap sampling methods used are biased towards *Cryptotermes brevis* captures. *Kalotermes flavicollis* often occurs in trees (e.g. public parks) and subterranean species may not produce alates; if they do, these can be difficult to distinguish from *C. brevis*, although swarming seasons differ.

## Geographic coverage

### Description

This study covered all Azorean islands, but termites were found only in six islands, from west to east: Faial, Pico, São Jorge, Terceira, São Miguel and Santa Maria.

### Coordinates

36.938 and 38.779 Latitude; -28.643 and -25.015 Longitude.

## Taxonomic coverage

### Description

Four species of termites (Insecta, Blattodea) were surveyed: the West Indian drywood termite, *Cryptotermes brevis* (Walker, 1853); the yellow-necked drywood termite, *Kalotermes flavicollis* (Fabricius, 1793); the Western European subterranean termite, *Reticulitermes grassei* Clément, 1978; and the eastern subterranean termite, *Reticulitermes flavipes* (Kollar, 1837).

## Temporal coverage

### Notes

2011-02-16 / 2024-12-27

## Usage licence

### Usage licence

Creative Commons Public Domain Waiver (CC-Zero)

## Data resources

### Data package title

Monitoring Termites in the Azores Archipelago: A Comprehensive Dataset (2011–2024)

### Resource link

http://ipt.gbif.pt/ipt/resource?r=termites_scit

### Alternative identifiers

https://www.gbif.org/dataset/781731ba-9e43-434a-9950-38ac37b3959f

### Number of data sets

1

### Data set 1.

#### Data set name

Occurrence Table

#### Data format

Darwin Core Archive

#### Character set

UTF-8

#### Download URL

http://ipt.gbif.pt/ipt/resource?r=termites_scit

#### Data format version

1.2

#### Description

The dataset was published in the Global Biodiversity Information Facility platform, GBIF (Borges et al. 2025). The following data-table includes all the records for which a taxonomic identification of the species was possible. The dataset submitted to GBIF is structured as a sample occurrence dataset that has been published as a Darwin Core Archive (DwCA), which is a standardised format for sharing biodiversity data as a set of one or more data tables. The core data file contains 1832 records (eventID; and occurrenceID). This GBIF IPT (Integrated Publishing Toolkit, Version 2.5.6) archives the data and, thus, serves as the data repository. The data and resource metadata are available for download in the Portuguese GBIF Portal IPT (Borges et al. 2025).

**Data set 1.** 

Column label	Column description
type	The nature of the record, typically used to distinguish Darwin Core Archives (in this case "PhysicalObject").
licence	A legal document giving official permission to do something with the data.
institutionID	An identifier for the institution having custody of the item.
datasetID	An identifier for the dataset from which the record was derived.
institutionCode	The code (often alphanumeric) for the institution having custody of the item (in this case UAc).
datasetName	The name of the dataset from which the record was derived.
basisOfRecord	The specific nature of the data record (in this case “HumanObservation”), a subtype of dcterms:type; recommended to use the Darwin Core Type Vocabulary. dwc.tdwg.orgdocs.google.com.
occurrenceID	An identifier for the Occurrence (as opposed to the specimen, observation or other data).
recordedBy	A list of names (concatenated and separated) of people, teams or organisations who recorded the original Occurrence.
establishmentMeans	The process by which the organism(s) represented became established at the location (e.g. “native”, “invasive”); controlled vocabulary defined by the TDWG establishmentMeans list dwc.tdwg.org.
occurrenceRemarks	Comments or notes about the Occurrence.
eventID	An identifier for the sampling event (as distinct from the occurrence).
eventDate	The date-time or interval during which the event occurred; in YYYY-MM-DD or ISO 8601 format.
year	The four-digit year in which the event occurred.
month	The integer month in which the event occurred (1–12).
day	The integer day of the month on which the event occurred (1–31).
habitat	A description of the habitat in which the event occurred.
samplingProtocol	The name of, reference to, or description of the method or protocol used during a sampling event.
sampleSizeValue	A numeric value for the size (e.g. count or weight) of the sample.
sampleSizeUnit	The unit of measurement for sampleSizeValue (e.g. days or hours).
samplingEffort	The amount of effort expended during a dwc:Event.
locationID	An identifier for the location.
continent	The name of the continent in which the Location occurs.
islandGroup	The name of the island group in which the Location occurs.
island	The name of the island on or near which the Location occurs.
country	The name of the country or major administrative unit in which the Location occurs.
countryCode	The ISO 3166-1-alpha-2 country code for the country in which the Location occurs.
municipality	The next smaller administrative region than county (e.g. city, town).
locality	The specific description of the place.
verbatimLocality	The original textual description of the place.
minimumElevationInMetres	The lower limit of the range of elevation.
decimalLatitude	Latitude in decimal degrees, using the spatial reference system given in geodeticDatum.
decimalLongitude	Longitude in decimal degrees, using the spatial reference system given in geodeticDatum.
geodeticDatum	The ellipsoid, geodetic datum or spatial reference system (SRS) upon which the geographic coordinates given in dwc:decimalLatitude and dwc:decimalLongitude are based.
coordinateUncertaintyInMetres	The horizontal distance (in metres) describing the uncertainty of the coordinates.
coordinatePrecision	The precision of the coordinates, given as the number of decimal places.
georeferenceSources	A list of sources (literature, URLs) for the georeference.
identifiedBy	A list of names (concatenated and separated) of people who determined the taxonomic identification.
dateIdentified	The date on which the identification was made.
identificationRemarks	Comments or notes about the taxonomic identification.
scientificName	The full scientific name, with authorship, of the lowest taxon rank.
kingdom	The full scientific name of the kingdom in which the taxon is classified.
phylum	The full scientific name of the phylum in which the taxon is classified.
class	The full scientific name of the class in which the taxon is classified.
order	The full scientific name of the order in which the taxon is classified.
family	The full scientific name of the family in which the taxon is classified.
genus	The full scientific name of the genus in which the taxon is classified.
specificEpithet	The name of the first or species epithet of the scientificName.
taxonRank	The taxonomic rank of the most specific name in the scientificName (e.g. “species”, “subspecies”).
scientificNameAuthorship	The person(s) and year attribution for the scientificName.

## Additional information

The four termite species were sampled during the survey period. Records of *Cryptotermes brevis* overwhelmingly dominated the monitoring dataset, accounting for 1,801 out of 1,832 total detections (98%). This striking prevalence aligns with previous findings that highlight the species' rapid and persistent expansion within urban environments across the Azores (Ferreira et al. 2013, Borges et al. 2014, Guerreiro et al. 2014). The vast majority of records were concentrated on two islands: Terceira (n = 919) (Fig. 2) and São Miguel (n = 755) (Fig. 3). In contrast, Faial, Pico and Santa Maria each yielded approximately 40 records, while São Jorge reported only seven detections.

Annual trap-capture counts across the archipelago exhibited a consistent upward trend, increasing from around 40 in 2011 to 154 in 2024, with a notable peak of 185 in 2023. These figures reflect the ongoing establishment and range expansion of *C. brevis*.

The yellow-necked drywood termite, *K. flavicollis*, was the second most frequently recorded species, with 24 detections, primarily on São Miguel, indicating a more localised and limited distribution pattern. The two subterranean termite species, *Reticulitermes grassei* and *R. flavipes*, were detected exclusively on Faial and Terceira, respectively. These findings are consistent with their previously known, geographically restricted distributions within the archipelago (Austin et al. 2012, Duarte et al. 2018).

The most recent government monitoring efforts conducted in 2022 and 2023 (Secretaria Regional do Ambiente e Alterações Climáticas 2023, Secretaria Regional do Ambiente e Ação Climática 2024) that support this manuscript have revealed a significant expansion in the distribution of *Cryptotermes brevis*, across several islands in the Azores Archipelago. These findings highlight the continued spread of this highly destructive urban pest into new localities, reinforcing the need for coordinated monitoring and control measures.

Based on the data collected in 2022, that was the first whole Azores termite survey so far implemented, new infestations were confirmed in several places previously considered unaffected. These areas were consequently added to the official registry requiring termite inspection certification for property transactions.

Pico Island: Lajes do Pico

Terceira: Serreta, Feteira and Ribeirinha (Angra do Heroísmo)

São Miguel Island - Rabo de Peixe and Matriz (Ribeira Grande), Vila Franca do Campo, São Vicente Ferreira (Ponta Delgada)

The data from 2023 report an even broader expansion in several islands:

Terceira: Terra-Chã, Ribeirinha, Vila de São Sebastião (Angra do Heroísmo)

Santa Maria: Almagreira (Vila do Porto)

These newly-identified localities mark a clear and ongoing range expansion of *C. brevis*, highlighting its invasive capacity and the urgency of applying integrated pest management strategies throughout the affected urban landscapes in the Azores.

Considering the trends in SCIT records of *C. brevis* in Terceira and São Miguel Islands (Fig. 4), the trend is similar to both islands, showing a sharp increase in records starting in 2015, peaking between 2017 and 2019, followed by a gradual decline through 2024.

## Figures and Tables

**Figure 1a.**
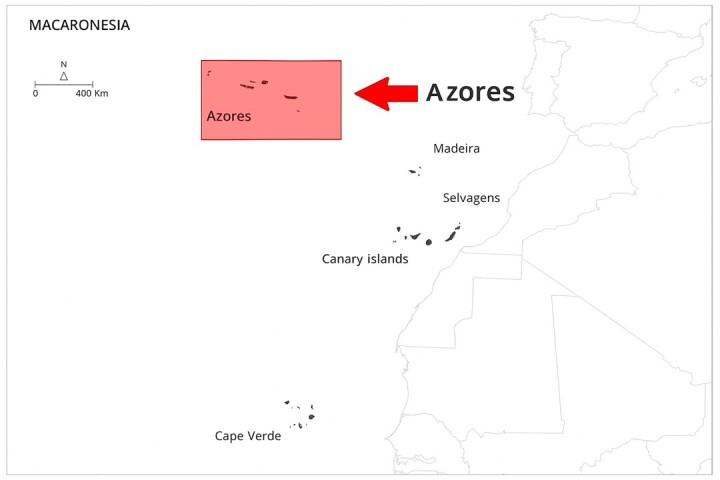


**Figure 1b.**
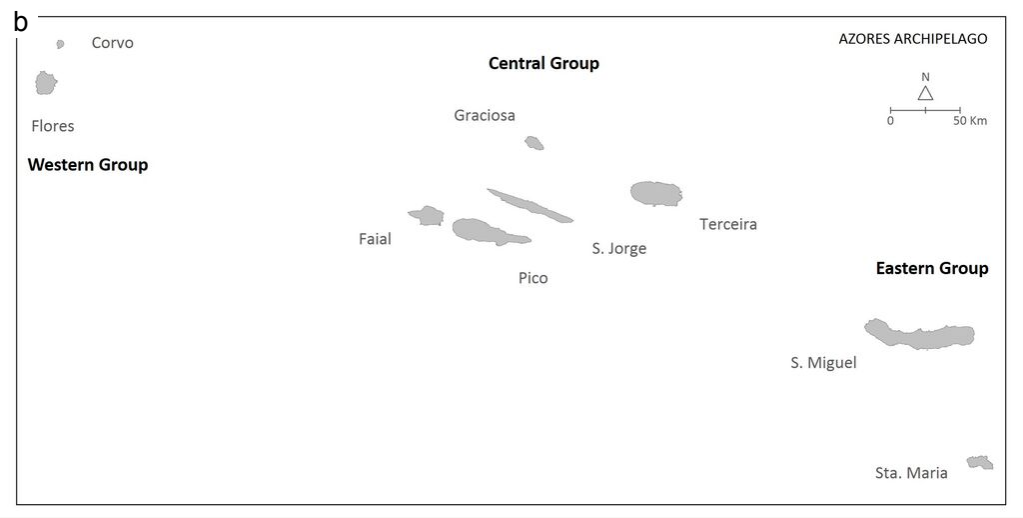


**Figure 2a.**
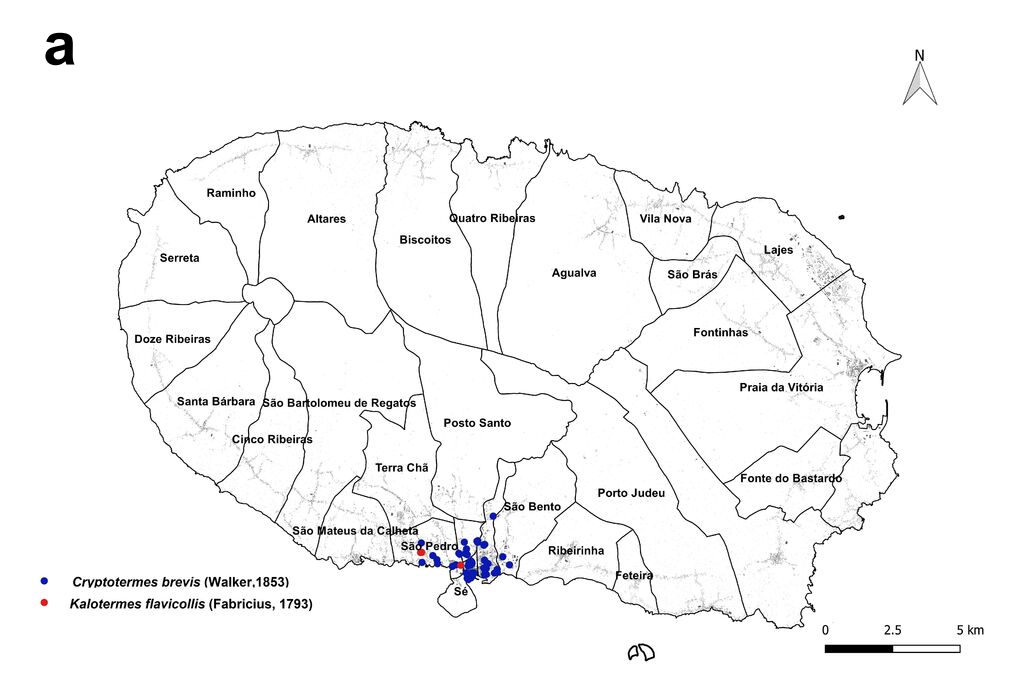
Whole island trap data points;

**Figure 2b.**
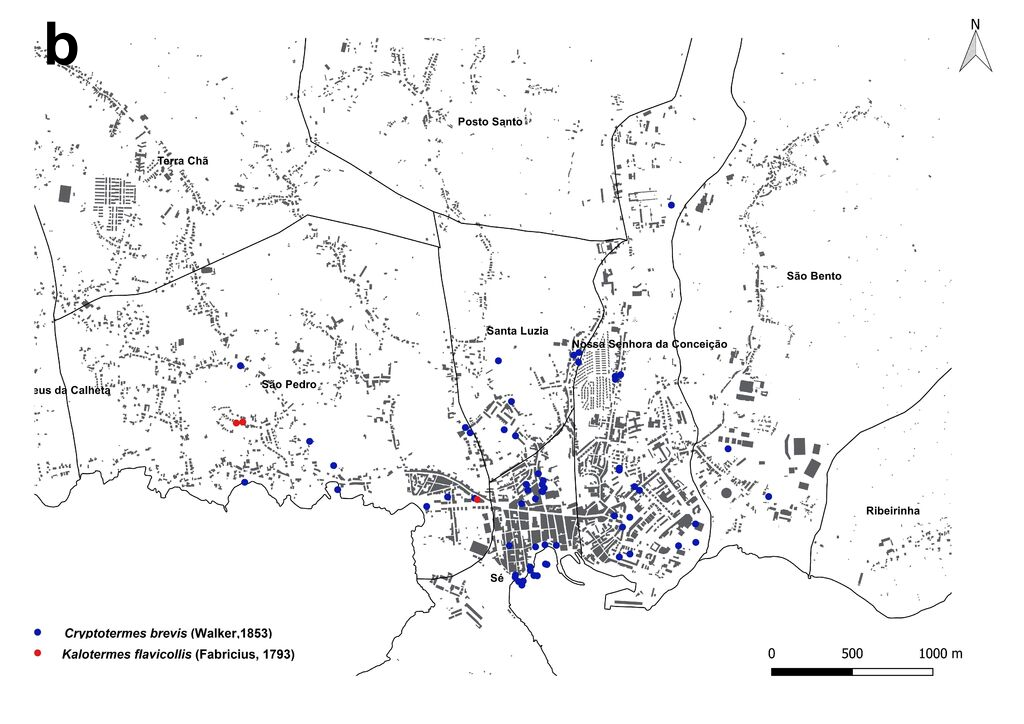
Main town of Angra do Heroísmo trap data points;

**Figure 2c.**
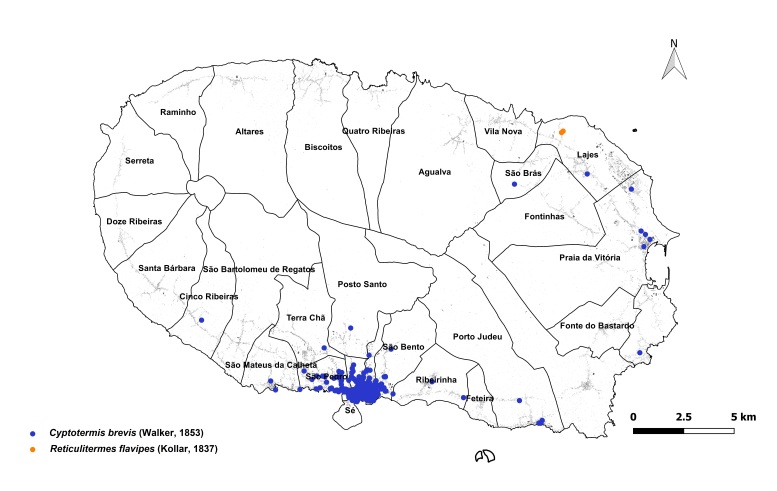
Whole island SCIT data points;

**Figure 2d.**
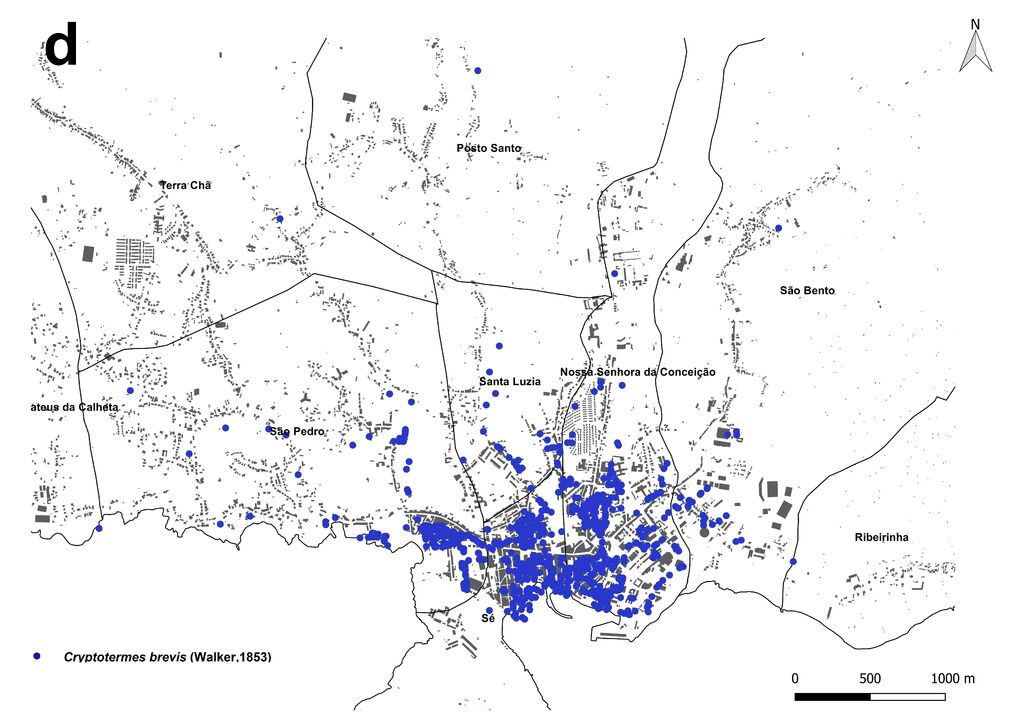
Main town of Angra do Heroísmo SCIT data points.

**Figure 3a.**
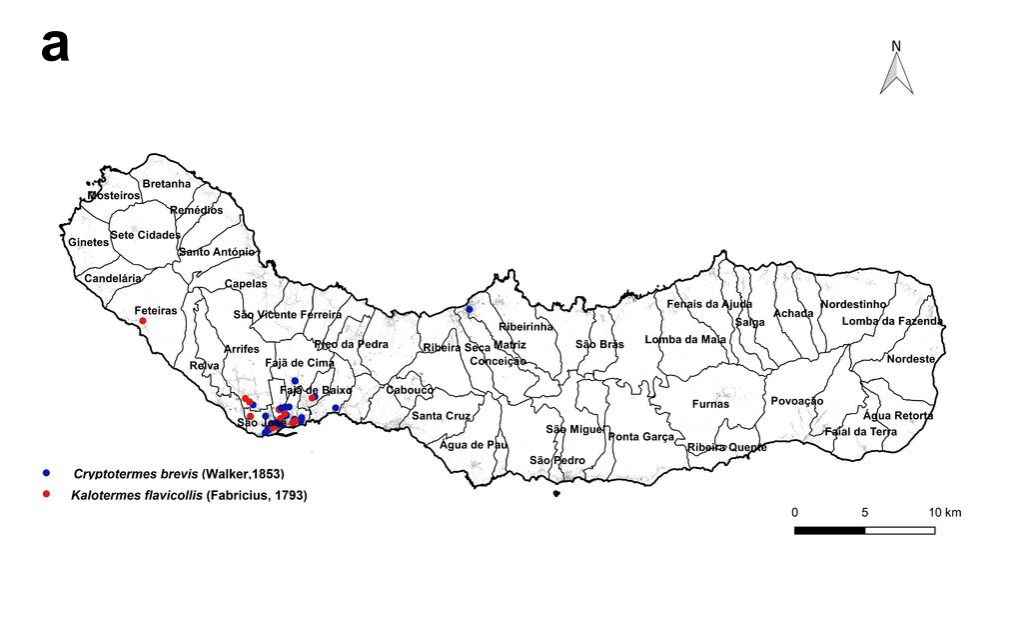
Whole island trap data points;

**Figure 3b.**
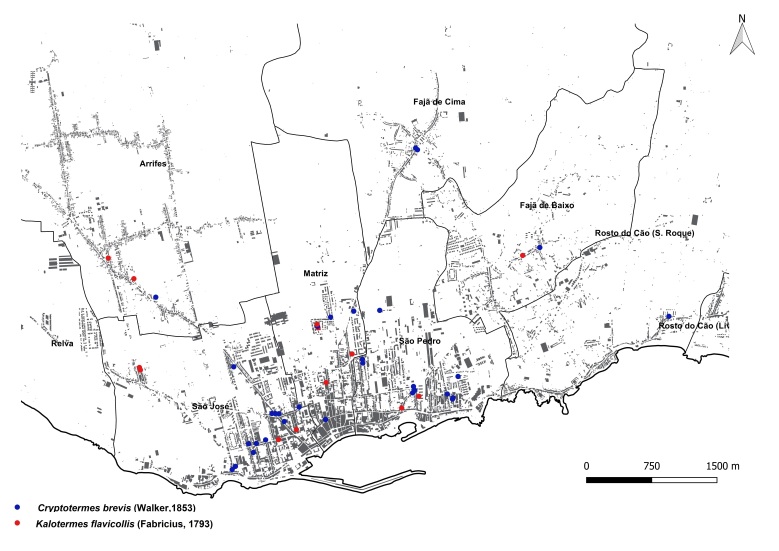
Main town of Ponta Delgada trap data points;

**Figure 3c.**
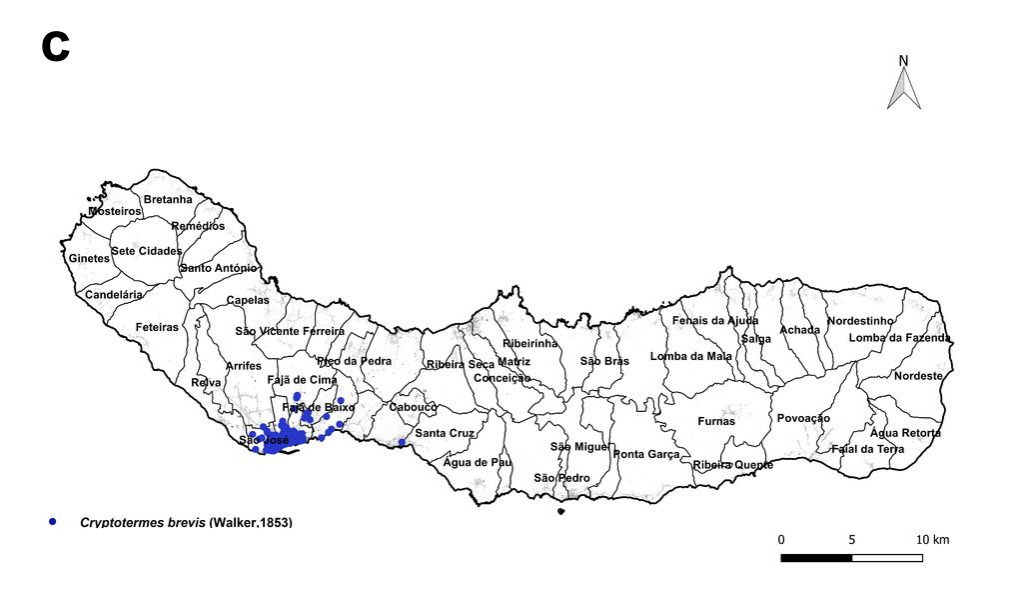
Whole island SCIT data points;

**Figure 3d.**
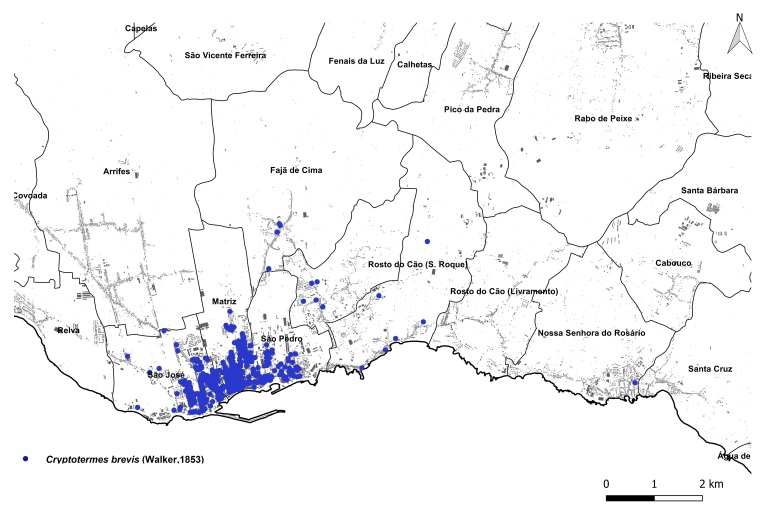
Main town of Ponta Delgada SCIT data points.

**Figure 4a.**
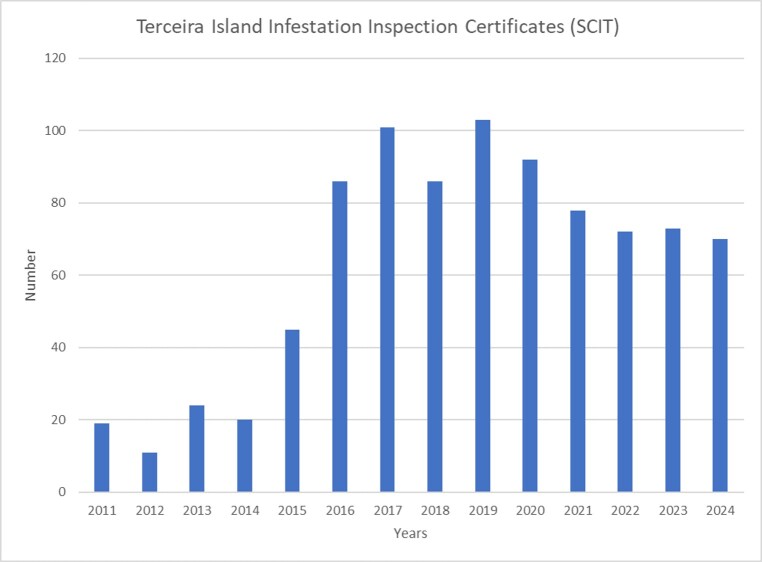
Terceira Island SCIT records;

**Figure 4b.**
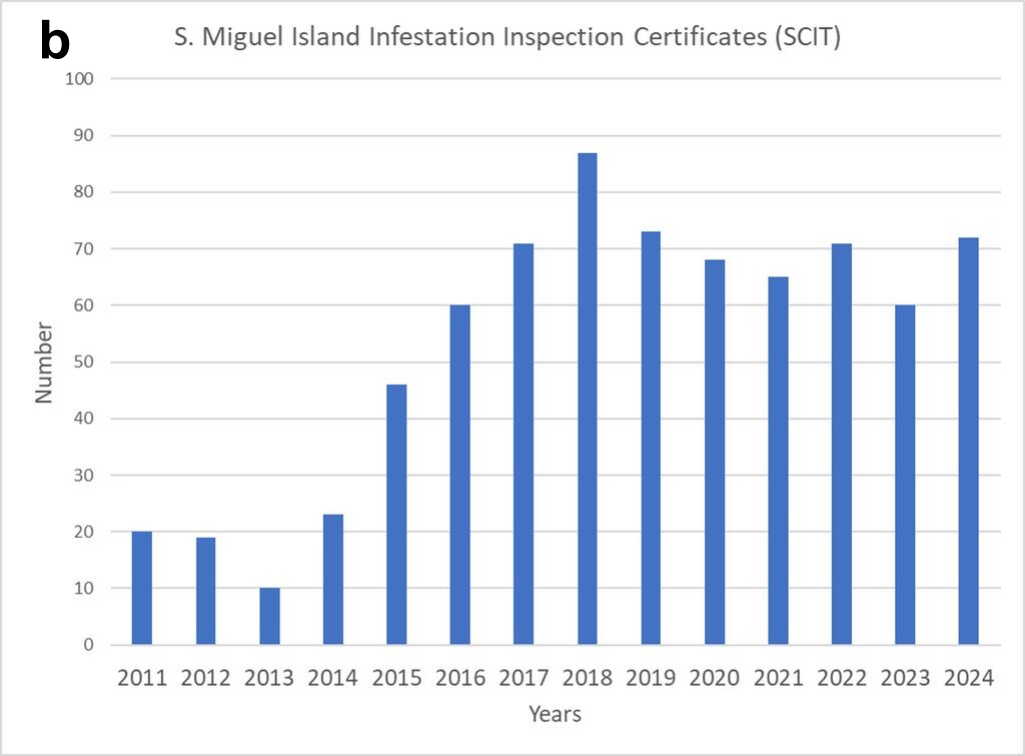
São Miguel Island SCIT records.
